# Neutrophil extracellular traps induced by VP1 contribute to pulmonary edema during EV71 infection

**DOI:** 10.1038/s41420-019-0193-3

**Published:** 2019-07-04

**Authors:** Nan Wang, Xiaofan Yang, Jiandong Sun, Zhixiao Sun, Qiyun Ma, Zhengxia Wang, Zhongqi Chen, Zibin Wang, Fan Hu, Huijuan Wang, Linfu Zhou, Mingshun Zhang, Juan Xu

**Affiliations:** 1 Department of Immunology, Nanjing Medical University, 210016 Nanjing, China; 2 NHC Key Lab of Antibody Technique, Nanjing Medical University, 210016 Nanjing, China; 3 Department of Respiratory and Critical Care Medicine, The First Affiliated Hospital of Nanjing Medical University, 210029 Nanjing, China; 4 The Laboratory Center for Basic Medical Sciences, Nanjing Medical University, 210016 Nanjing, China; 5 Department of Infectious Disease, Nanjing Medical University Nanjing First Hospital, 210006 Nanjing, China; 6 Analysis center, Nanjing Medical University, 210016 Nanjing, China; 7 State Key Laboratory of Reproductive Medicine, Nanjing Medical University, 210016 Nanjing, China

**Keywords:** Immune cell death, Viral infection

## Abstract

Pulmonary edema is a fatal complication of EV71-associated hand, foot, and mouth disease (HFMD). The pathogenesis of EV71-induced pulmonary edema remains largely unclear. In this study, we aimed to explore the roles of the capsid protein VP1 in the occurrence of EV71-induced pulmonary edema. The intranasal inoculation of recombinant VP1 protein caused lung inflammation with an elevation of inflammatory cytokines and neutrophils infiltration. Moreover, neutrophil extracellular traps (NETs) were observed in the lung parenchyma of the mice treated with VP1. VP1 directly induced the formation of NETs, which depended on PAD4. VP1 also damaged the lung barrier via the reduction of the tight junction protein occludin. Moreover, the EV71 attachment receptor vimentin was increased upon VP1 administration. In contrast, NETs decreased vimentin levels, suggesting a novel role for NETs in viral immune defense. These results evidenced a direct role of VP1 in EV71-induced pulmonary edema and demonstrated that NETs may be both harmful and beneficial in EV71 infection.

## Introduction

Hand, foot, and mouth disease (HFMD) is an infectious disease that affects millions of young children and infants worldwide^1^. Enterovirus 71 (EV71) is considered as the major cause of HFMD. Although most HFMD is self-limiting and is a mild illness, EV71 infection may cause serious neurological, cardiac, and respiratory problems in young children^2^. Pulmonary edema is considered as the major cause of HFMD-related fatalities. It is postulated that the occurrence of pulmonary edema is associated with CNS lesions^3^. However, the pathogenesis of EV71-induced pulmonary edema is still largely unclear.

In a previous study, EV71 mRNAs were detected in lung tissue, providing evidence for EV71 replication in lung tissue^4^. Further, EV71 antigen was widely distributed in lung tissue both in fatal EV71 patients and in an EV71-infected mouse model^5^. VP1 is acapsid protein responsible for EV71 genotyping^6^ and virus entry^7^. We hypothesize that the presence of VP1 in the lung tissue could be involved in lung injury. Therefore, we directly introduced recombinant VP1 protein into the lungs of mice and examined the inflammatory responses.

Neutrophil extracellular traps (NETs)^8^ are composed of nuclear chromatin, hypercitrullinated histones, granular antimicrobial proteins, and cytoplasmic proteins^9^. Similar to a double-edged sword, NETs may function in immune defenses, but they may also magnify inflammation^10^ in diverse diseases. Many types of viruses can induce NET formation^11–13^, and recent studies have revealed that excessive NET formation may cause adverse effects in a number of lung diseases, including acute lung injury^14–16^. We further evaluated whether VP1 could induce the formation of NETs in the lung.

Moreover, life-threatening pulmonary edema may be accompanied by the loss of epithelial integrity. Tight junctions and adherens junctions have barrier functions. ZO-1 and occludin are two of the best-characterized tight junction proteins^17–19^. In addition, E-cadherin is an important adherens junction protein^20^. Therefore, we explored the role of VP1 in the alterations of the lung epithelial barrier and junction proteins. The intermediate filament protein vimentin is a type of adherens junction protein. More importantly, vimentin contributes to EV71 virus entry^21^. Finally, we examined the roles of VP1 and NETs in the expression of vimentin.

## Results

### VP1-induced acute lung injury

**Fig. 1:**
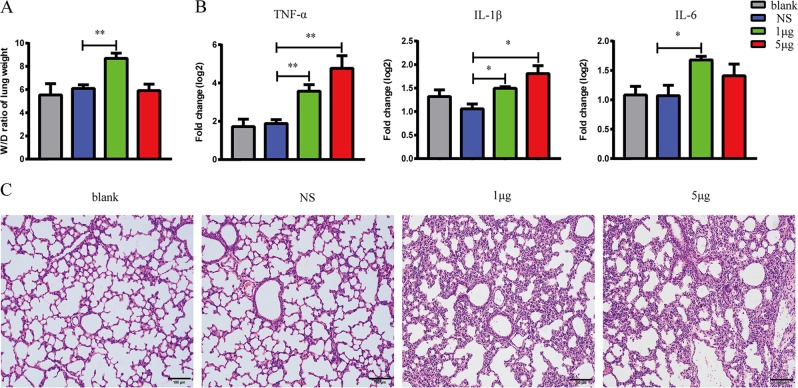
VP1-induced acute lung injury. **a** The effects of VP1 on pulmonary edema were determined by the wet-to-dry (W/D) lung weight ratio 4 h after VP1 intranasally administered. **b** Relative mRNA expression (log2) of inflammatory cytokines (TNF-α, IL-1β, and IL-6) in lungs was determined by qRT-PCR. **c** Representative images of H&E‐stained lung sections for the analysis of inflammatory cell infiltration and pulmonary edema; magnification, ×200; scale bar, 100 μm. The data are shown as the mean ± SEM. *n* = 3–5 per group. *p < 0.05, **p < 0.01, ***p < 0.0001

We introduced VP1 directly into mouse lung tissue by intranasal administration to evaluate whether VP1 could induce acute lung injury. Our study revealed that the wet-to-dry (W/D) ratio in the VP1 group was significantly higher than that of the blank and NS groups (Fig. 1a), which was indicative of pulmonary edema and of injury to the pulmonary epithelial barrier. Accordingly, the transcript levels of inflammatory cytokines (TNF-α, IL-1β, and IL-6) in mice treated with VP1 were significantly increased compared to the control groups (Fig. 1b). Moreover, hematoxylin and eosin (H&E) staining suggested that there was destruction in pulmonary epithelial architecture, an increase in inflammatory cell infiltration in the alveolar space and an increase in interstitial edema in the VP1-treated mice (Fig. 1c). In brief, these observations demonstrated that more severe lung injury occurred in the mice with VP1 exposure compared with that in the blank and saline groups.

### VP1 recruited neutrophils into the lung

**Fig. 2:**
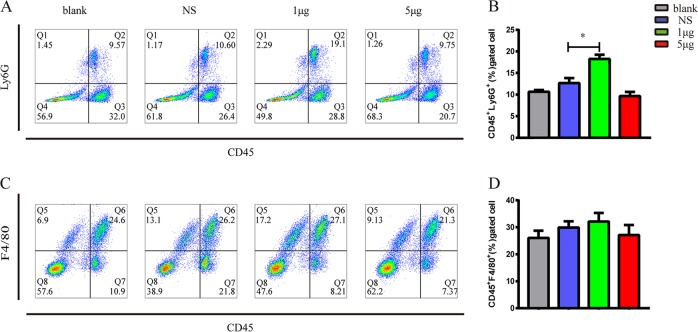
VP1 recruited neutrophils into the lung. The single-cell suspensions were obtained from the lungs of mice from each experimental group and stained with anti-CD45-FITC, anti-Ly6G-PE, and anti-F4/80-APC antibodies. Flow cytometric analysis was performed and representative flow cytometry pseudocolor plots gated on neutrophils (CD45^+^Ly6G^+^) and macrophages (CD45^+^F4/80^+^) were shown. **a**, **b** The percentage of neutrophils in the lung was markedly increased in VP1-treated mice. **c**, **d** The percentage of macrophages in the lung was slightly increased in VP1-treated mice. Values are expressed as the mean ± SEM. *n* = 4–6 per group. *p < 0.05, **p < 0.01, ***p < 0.0001

Further, we analyzed the types of inflammatory cells that had infiltrated into lung tissue after VP1 inhalation using flow cytometry. As shown in Fig. 2a, b, neutrophil infiltration increased in the 1 μg VP1-treated group compared to the saline group. Although VP1 caused an increase in macrophages compared with the control group, it was not statistically significant (Fig. 2c, d). In conclusion, VP1 promoted neutrophil infiltration into lung tissue.

### VP1 increased neutrophil chemokines in lung tissue

**Fig. 3:**
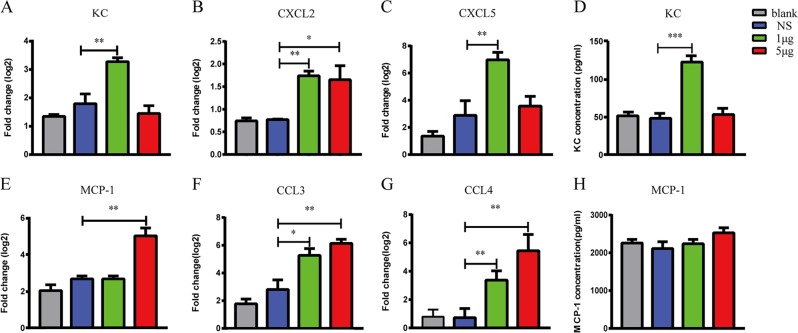
VP1 increased neutrophil chemokines in lung tissue at both the RNA and protein levels. Real-time PCR assays were performed to detect the mRNA expression levels of the neutrophil chemokines **a**–**c** KC, CXCL2, and CXCL5 and the macrophage chemokines **e**–**g** MCP-1, CCL3, and CCL4. ELISA assays were performed to detect the protein levels of **d** KC and **h** MCP-1 in lung homogenates. Data are presented as the mean ± SEM. *n* = 3–5 for each group. *p < 0.05, **p < 0.01, ***p < 0.0001

To further clarify the reasons for the increased neutrophils and macrophages infiltration, we examined neutrophil chemokines (KC, CXCL2, and CXCL5)^22–24^ and macrophage chemokines (MCP-1, CCL3, and CCL4)^25,26^ in lung from the mice that were or were not treated with VP1. As shown, the transcript levels of the neutrophil chemokines KC (Fig. 3a), CXCL2 (Fig. 3b), and CXCL5 (Fig. 3c) and the macrophage chemokines MCP-1 (Fig. 3e), CCL3 (Fig. 3f), and CCL4 (Fig. 3g) were significantly increased in the VP1-treated mice compared with that in the blank and saline groups. Furthermore, VP1 increased the KC protein expression in the lung homogenates (Fig. 3d). Consistent with the results shown in (Fig. 2d), there was no significant increase in the macrophage chemokines MCP-1 (Fig. 3h) in the VP1-treated group. In summary, neutrophils infiltrated into lung tissue in VP1-treated mice may at least partly depend on chemokines.

### VP1-induced neutrophil extracellular traps

**Fig. 4:**
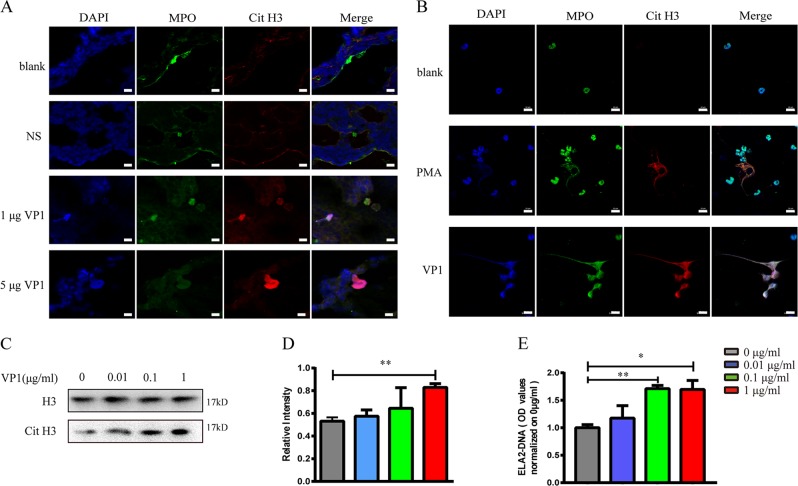
VP1-induced NET formation. **a** NETs in lung were stained and observed by confocal microscopy; magnification, ×400; scale bar, 10 µm. **b** The formation of NETs was observed by confocal microscopy in vitro; magnification, ×400; scale bar, 10 μm. **c**, **d** Citrullinated histone 3 was measured using western blotting. Grayscale analysis confirmed the repeated results. **e** The NE-DNA ELISA quantitatively detected NETs in the culture supernatant of neutrophils treated with different concentrations of VP1. Data are shown as the mean ± SEM. *n* = 3 for each group. *p < 0.05, **p < 0.01, ***p < 0.0001

We further explored whether VP1 could induce NET formation and subsequently lead to acute lung injury. To test this hypothesis, we detected citrullinated histones and myeloperoxidase (MPO) in lung tissues of each treatment group by immunofluorescence. As expected, NETs were detected in lung tissue of mice treated with VP1, which was demonstrated by the presence of extracellular DNA overlaid with citrullinated histone 3 and MPO (Fig. 4a). To determine whether VP1 may directly induce NETs, neutrophils that were isolated from the bone marrow of mice were stimulated with different concentrations of VP1. As shown in Fig. 4e, neutrophil elastase-DNA (NE-DNA) complex expression was significantly elevated in a concentration-dependent manner in the culture supernatant of neutrophils treated with VP1. Similarly, western blotting showed that citrullinated histone 3 also increased in a concentration-dependent manner (Fig. 4c, d). According to the NE-DNA ELISA and western blotting results shown above, 1 μg/ml VP1 was selected to stimulate NET formation, and NET formation was also observed by confocal microscopy; this was similar to stimulation by 100 nM phorbol-myristate acetate (PMA), which is commonly employed as a potent nonphysiologic agonist to induce NET formation in vitro^27,28^ (Fig. 4b). Collectively, VP1 directly promoted the formation of NETs that may further damage lung tissue.

### Dynamic observation of NET formation by a living-cell imaging technique

**Fig. 5:**
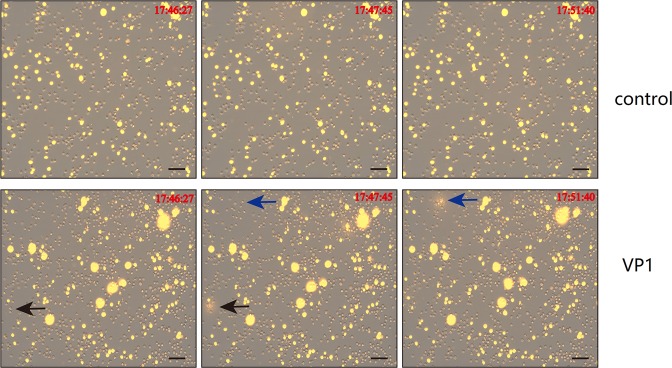
Dynamic observation of NET formation by a living-cell imaging technique. The time in the picture referred to the time when the data were collected by a Zeiss Cell Discoverer 7 microscope system; scale bar, 50 μm. Two arrows (black and blue) indicated the neutrophils undergoing NETs

To more directly observe NET formation, neutrophils with or without VP1 stimulation were placed into a live-cell imaging system for real-time observation^29,30^. SYTOX orange (1 μM) was added to each cell culture well before neutrophils were placed into a live-cell imaging system^31^. Images were acquired with a Cell Discoverer 7 live-cell imaging system (Carl Zeiss Microscopy) using an excitation wavelength of 558 nm, and detection was performed using an emission wavelength of 575 nm. We observed that NETs formed in the VP1-treated neutrophils (Fig. 5). In addition, NETs were first observed at ~90 min after VP1 stimulation (refer to the video in the supplementary materials for details)^32^.

### VP1 promotes NETosis via a PAD4-dependent pathway

**Fig. 6:**
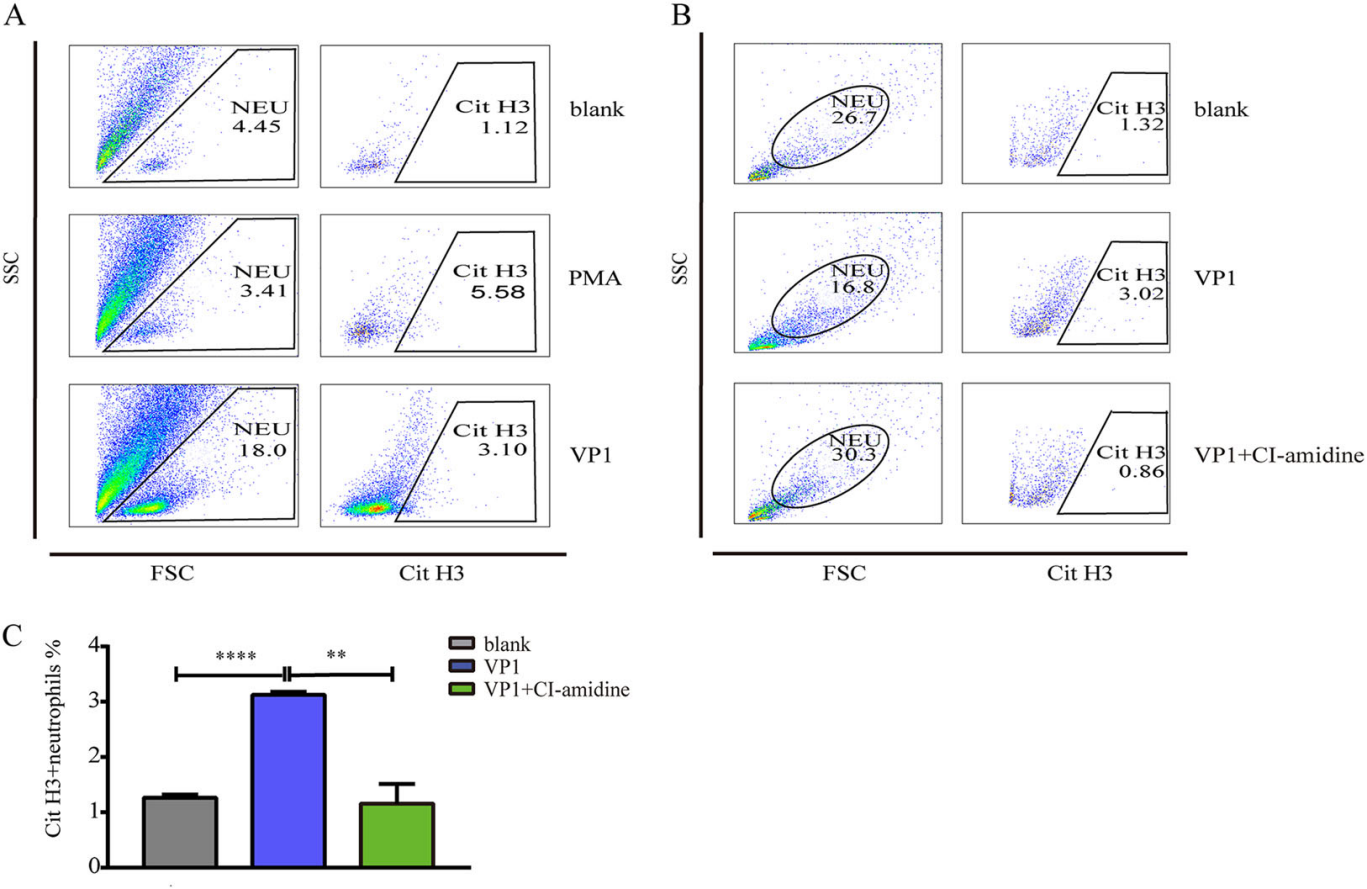
VP1 promoted NETs via the PAD4-dependent pathway. **a** Flow cytometry analysis revealed the percentage of citrullinated H3-positive neutrophils. **b**, **c** The PAD4 inhibitor Cl-amidine significantly decreased the percentage of citrullinated H3-positive neutrophils in the VP1-treated group. Data are shown as the mean ± SEM. *n* = 3 for each group. *p < 0.05, **p < 0.01, ***p < 0.0001

The peptidylarginine deaminase 4 (PAD4)-dependent histone citrullination and decondensation of chromatin have been regarded as a key step in the formation of NETs^33–35^. NETosis also depends on the generation of reactive oxygen species by NADPH oxidase^36,37^. However, an understanding of the effect of these enzymes on VP1 remains lacking. We used citrullinated histone 3 as a biomarker for neutrophil priming for NETosis, and we examined the levels of circulating CitH3^+^ cells and found that a greater percentage of neutrophils was primed for NETosis in the VP1-treated mice (Fig. 6a), which was second only to the levels observed in the positive control (PMA) group. To further evaluate how VP1 triggered NETosis, we added the PAD4 inhibitor Cl-amidine^38,39^ or the NADPH oxidase inhibitor diphenyleneiodonium (DPI)^40,41^ half an hour before VP1 stimulation. We found that Cl-amidine inhibited NET formation triggered by VP1 (Fig. 6b, c), whereas DPI did not (data not shown). In conclusion, VP1 promoted NET formation in a PAD4-dependent and NADPH oxidase-independent manner.

### VP1 directly damages lung tissue barrier function

**Fig. 7:**
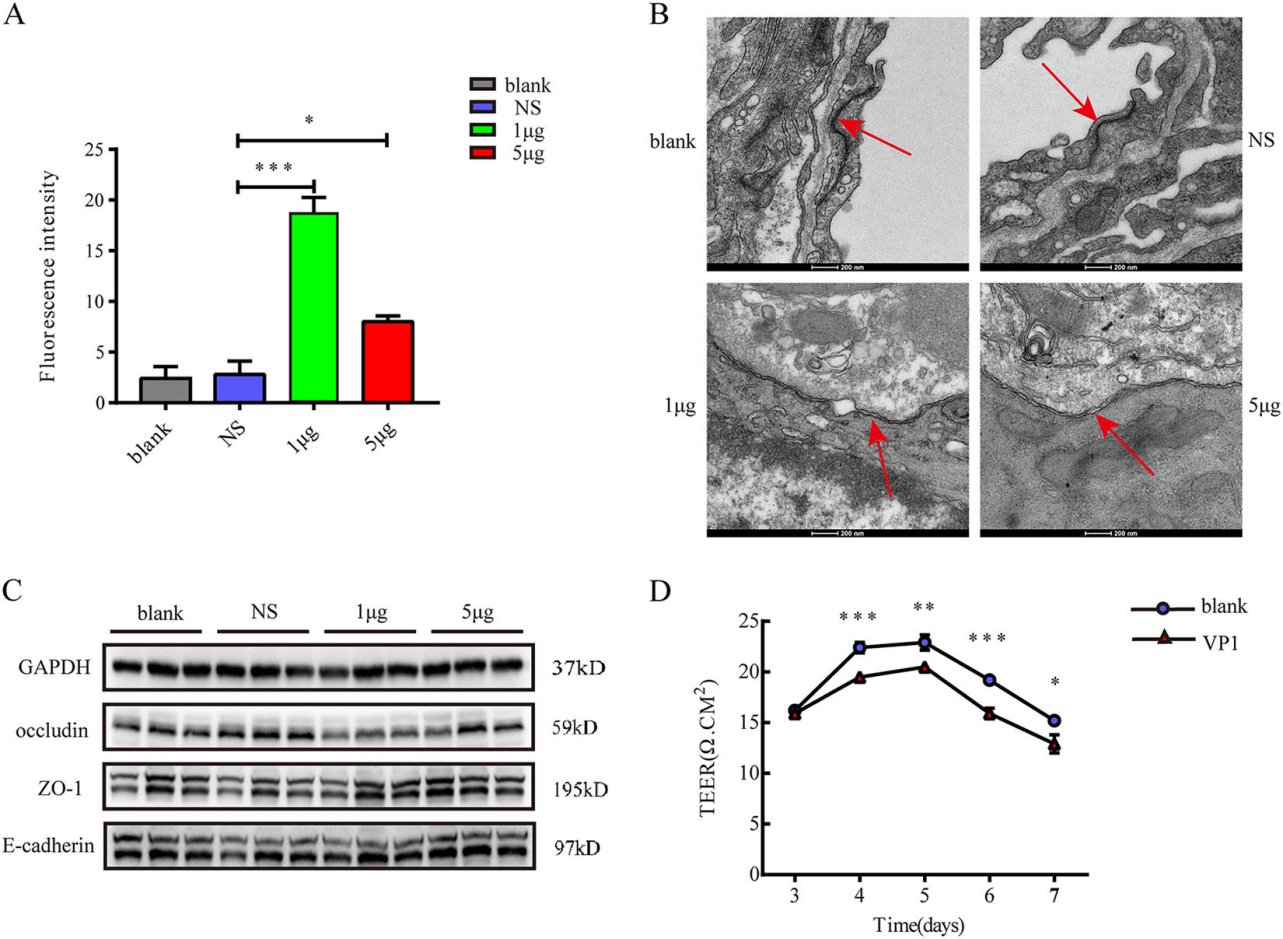
VP1 directly damaged lung tissue barrier function. **a** Bronchoalveolar epithelial permeability was determined by measuring the leakage of FD4 in lung. Data are presented as the mean ± SEM. *n* = 3–5 for each group. **b** The integrity of the pulmonary epithelium and morphological alterations of junctions was studied by transmission electron microscopy in both VP1-treated and untreated groups. **c** The western blotting assay of lung tissue tight junction protein and adherens junction protein in each group. **d** The TEER of the MLE‐12 cellular monolayer over time treated or untreated with VP1. Data are expressed as the mean ± SEM, *n* = 3. *p < 0.05, **p < 0.01, ***p < 0.0001

Previous results confirmed that VP1 promoted the infiltration of neutrophils into lung tissue by increasing the chemokine levels of neutrophils and indirectly damaged lung tissue by promoting the formation of NETs. Next, we explored whether VP1 could directly damage the pulmonary epithelial barrier function both in vivo and in vitro. The bronchoalveolar epithelial permeability was determined by measuring the leakage of FD4 from the blood vessels into the lung. As shown in Fig. 7a, the fluorescence density of VP1-treated mice was significantly higher than that of the blank group and normal saline group. Since VP1 treatment increased the bronchoalveolar epithelial permeability in mice, it was likely to change the morphology of the junctions in the alveolar epithelium. The integrity of the pulmonary epithelium barriers and the morphological alterations of the junctions were studied by transmission electron microscopy in mice with or without VP1 treatment. The random sampling of the left lung from the blank and NS groups showed intact junctions between all of the epithelial cells. In contrast, after exposure to VP1 for 4 h, the random sampling of the left lungs from VP1-treated mice showed the loss of junctions between the alveolar epithelial cells. The junctions were irregularly widened (Fig. 7b). The protein expression levels of occludin were decreased in the VP1 group compared to the blank and NS groups. In contrast, there was no significant change in the protein levels of ZO-1 and E-cadherin in each treatment group (Fig. 7c). In vitro transepithelial electrical resistance (TEER) measurements were carried out to evaluate the integrity of the MLE-12 cellular monolayers^42,43^. VP1 significantly decreased the TEER and epithelial integrity of the pulmonary epithelial cells. (Fig. 7d). Collectively, VP1 may damage the pulmonary epithelial barrier function via the downregulation of tight junction protein occludin.

### VP1 increased vimentin, while NETs downregulated vimentin on the pulmonary epithelial cells

**Fig. 8:**
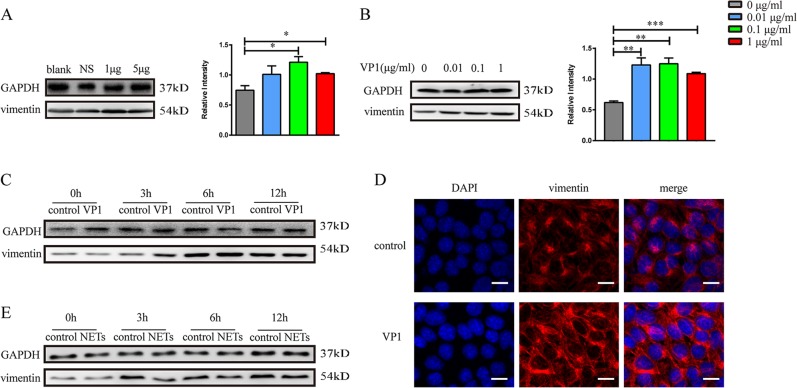
VP1 upregulated vimentin expression, while NETs downregulated vimentin expression. **a** Western blotting analysis of vimentin protein in lung tissue in each group. **b**, **c** Role of VP1 on the vimentin protein in MLE-12. **d** The confocal microscopy assay of vimentin protein in MLE-12. **e** The effect of NETs on the expression of vimentin in MLE-12. Data are shown as the mean ± SEM. *n* = 3 for each group. *p < 0.05, **p < 0.01, ***p < 0.0001

The junction protein vimentin is an attachment receptor for EV71^21^. Intranasal introduction of VP1 increased the expression of vimentin in lung tissue (Fig. 8a). To further explore whether vimentin could be augmented on the lung epithelial cells by VP1, MLE-12 cells were stimulated with recombinant VP1 protein. As shown in Fig. 8b–d, VP1 increased vimentin expression in the lung epithelial cells. Interestingly, vimentin was downregulated when NETs acted directly on the MLE-12 cells (Fig. 8e). Of note, vimetin in the control group without stimulus seemed increased during the time course (Fig. 8c, e), which was in the line with the observation that epithelial cells growth and migration may cause the elevation of vimetin^44^. In summary, VP1 may positively promote the virus receptor vimentin, which was negatively regulated by NETs.

## Discussion

Pulmonary edema is a major cause of EV71-associated mortality. Some studies have shown that compared with EV71 patients with uncomplicated brainstem encephalitis, patients with brainstem encephalitis accompanied by pulmonary edema have significantly higher serum and CSF levels of proinflammatory cytokines^45,46^. In addition, inflammatory cells (neutrophils, macrophages, mast cells, etc.) were demonstrated to contribute to EV71 pulmonary edema^4,47^. VP1 is the major virulence determinant of EV71. However, the pathological roles of VP1 in the initiation and progression of pulmonary edema remain largely elusive. Intranasal inoculation of recombinant VP1 protein promoted lung inflammation that was characterized by elevated inflammatory cytokines (TNF-α, IL-1β, and IL-6) and by neutrophils in the lung parenchyma. The increase in the chemokine KC may promote neutrophil infiltration into the lung, which was in accordance with previous findings that showed that neutrophils were widely present in the tissues and organs of patients infected with EV71^4^. Of note, VP1 at 1 μg seemed more potent that VP1 at 5 μg in the mouse model of acute lung injury, we observed that 5 μg VP1 diluted in 10 μl NS was a little viscous, which may cause the inadequate inhalation in the intranasal instillation and the consequently reduced effects. Still, our observations suggested that pulmonary edema might be the result not only of the direct hydrostatic force of systemic vasoconstriction resulting from brainstem injury but also of an increased pulmonary inflammatory response.

Although they were first identified in bacterial infections, NETs are also involved in viral infections^48^. The roles of NETs in EV71 infection, however, have not been demonstrated before. In the lung parenchyma of VP1-treated mice, we observed the formation of NETs. Interestingly, VP1 may directly trigger the formation of NETs, which was at least partially dependent on PAD4. Considering that different stimuli, including inflammatory cytokines, may trigger the formation of NETs, we could not preclude the possibility that EV71 may directly (via VP1) or indirectly (via inflammatory cytokines) promote the formation of NETs.

Damage to pulmonary permeability may cause fatal lung edema. As the W/D ratio increased and the fluorescence dextran leakage increased in the VP1-treated lung parenchyma, the pulmonary permeability became damaged, which was directly observed using transmission electron microscopy. The tight junction protein occludin was involved with lung integrity, and an occludin reduction may be accompanied by lung edema^49^. Accordingly, occludin was significantly decreased in the lungs of VP1-treated mice. In contrast to the tight junction protein occludin, the adherens junction protein vimentin mediates EV71 virus entry^21^. VP1 increased the expression of vimentin, suggesting that EV71 may amplify the infection with the increase in the virus receptor. Interestingly, VP1-induced NETs caused the downregulation of vimentin and, therefore, may alleviate EV71 infection. Therefore, NETs may not only damage the lung barriers as observed in the acute lung injury induced by other stimuli but may also contribute to the immune defense via the downregulation of virus receptors. Recently, it was shown that NETs may awaken dormant cancer cells via the cleavage of matrix proteins^50^. The mechanisms of NETs degrading vimentin warrant further study.

In conclusion, our study revealed that the EV71 capsid protein VP1 directly caused neutrophil infiltration and NET formation, which damaged the lung barrier and caused pulmonary edema. Moreover, NETs reduced expression of the virus receptor vimentin, which may represent a novel function of NETs in the viral immune defense.

## Materials and methods

### Animals and ethics statement

Wild type C57BL/6J mice (1 week old) that were maintained under specific pathogen-free conditions were obtained from the College of Veterinary Medicine of Yangzhou University (Yangzhou, China). The room temperature and humidity were set at 23 ± 3 °C and 55.5 ± 10%, respectively. All mice were maintained on a 12-h light–dark cycle. All animal protocols were reviewed and approved by the Animal Care and Use Committee of the Nanjing Medical University (1708004).

### VP1-induced acute lung injury

VP1 recombinant protein with low endotoxin (<0.1 EU/μg) was obtained from Genscript (Nanjing, China). Experimental animals were randomly divided into four groups: the blank control group (blank), saline group (NS), 1 μg VP1 group (1 μg), and 5 μg VP1 group (5 μg). The blank control group was untreated. The NS and VP1 groups were given NS droplets or NS droplets containing VP1 (1 μg or 5 μg of protein diluted in 10 μl of NS) intranasally after anesthetization by intraperitoneal injection with a mixture of 10 mg/kg xylazine (MTC Pharmaceuticals, Cambridge, ON, Canada) and 200 mg/kg ketamine hydrochloride (Rogar/STB, London, ON, Canada).

### Lung histopathological examination

Four hours after the last inoculation with saline or VP1, PBS was pumped into the right ventricle to clear the blood in the pulmonary vasculature. Then, the left lower lung lobe was excised, fixed in 4% paraformaldehyde for 24 h, embedded in paraffin, cut into 5 μm sections on a Leica model 2165 rotary microtome (Leica, Nussloch, Germany), and stained with H&E. For each animal, five fields were measured randomly from the H&E staining at a magnification of ×200.

### Wet/Dry lung weight ratio measurement

The right upper lung lobes from each group were excised at the end of the experiment and weighed to determine the wet lung weight. The tissues were then placed in a metal bath and incubated at a temperature of 80 °C for 24 h before being weighed again to give the final dry weight. The presence of edema was determined by calculating the pulmonary W/D ratio^51,52^.

### Measurement of bronchoalveolar epithelial permeability

To measure the bronchoalveolar epithelial permeability, a fluorescein isothiocyanate-conjugated dextran 4000 (FD4, Sigma-Aldrich, Bornem, Belgium) solution in PBS (25 mg/ml; 10 mg/kg) was injected intraperitoneally. Four hours later, after anesthesia and pulmonary perfusion, the fluorescence intensity of FD4 in homogenized lung tissue was measured using a fluorescence plate reader (Biotek synergy) at an excitation wavelength of 485 nm and an emission wavelength of 528 nm^53,54^.

### Transmission electron microscopy

After dissection of the lung, the specimens were fixed overnight in 2.5% glutaraldehyde at 4 °C. Then, the tissues were washed, fixed, dehydrated, and embedded. Random 60-nm transverse sections were collected on single copper slot grids coated with parlodion, stained with uranyl acetate and, lead citrate and observed with an FEI Tecnai G2 Spirit Bio TWIN transmission electron microscope.

### Flow cytometry

To prepare single cells from whole lungs, lung tissue was ground in PBS containing 1% BSA. Freed cells were washed with PBS containing 1% BSA and filtered using 70-mm nylon mesh. Cells (10^6^/100 µl) were blocked with 1 µl of purified anti-mouse CD16/CD32 (14-0161-85, Thermo) (FcR block) antibody for 10 min on ice to prevent nonspecific staining and then stained in the dark with a combination of anti-mouse CD45-FITC (MCD4501, Thermo), anti-mouse Ly6G-PE (12-5931-85, Thermo), and anti-mouse F4/80 antigen APC (17-4801-82, Thermo) at room temperature for 1 h. The stained cells were washed three times with ice-cold PBS and analyzed with a BD FACS ARIA II SORP (Special Order Research Product) flow cytometer with FlowJo_V10 software.

### RNA isolation and quantitative real-time PCR

Total RNA was obtained from fresh lung tissue with a TRIzol reagent kit (Life Technologies) and reverse-transcribed into cDNA with a reverse-transcription kit (Abm, Zhenjiang, China) according to the manufacturer’s instructions. TNF-α, IL-1β, IL-6, KC, CXCL2, CXCL5, CCL3, CCL4, MCP-1, and GAPDH mRNA expression was detected by a StepOnePlus Real-Time PCR System (ABI, USA). The primer sequences used for real-time PCR were designed by referring to PrimerBank (https://pga.mgh.harvard.edu/primerbank). The primers for those genes are shown in Table 1. Relative levels were determined using the 2^–ΔΔCt^ method, and GAPDH was used as the internal control^55^. Each sample was run in triplicate, and the results were representative of at least three independent experiments.

**Table 1:** Primers used in the study

Gene ID	Forward primer	Reverse primer
GAPDH	AGGTCGGTGTGAACGGATTTG	TGTAGACCATGTAGTTGA GGTCA
TNF-α	TCTCATCAGTTCTATGGCCC	GGGAGTAGACAAGGTACAAC
IL-1β	AGCTTCAAATCTCGCAGCAG	TCTCCACAGCCACAATGAGT
IL-6	GGCGGATCGGATGTTGTGAT	GGACCCCAGACAATCGGTTG
KC	CAAGGCTGGTCCATGCTCC	TGCTATCACTTCCTTTCTGTTGC
CXCL2	CCAACCACCAGGCTACAGG	GCGTCACACTCAAGCTCTG
CXCL5	GTTCCATCTCGCCATTCATGC	GCGGCTATGACTGAGGAAGG
CCL3	TTCTCTGTACCATGACACTCTGC	CGTGGAATCTTCCGGCTGTAG
CCL4	TTCCTGCTGTTTCTCTTACACCT	CTGTCTGCCTCTTTTGGTCAG
MCP-1	GTTGGCCTCAGCCAGATGCA	AGCGTACTCATTGGGATCATCTTG

### Enzyme-linked immunosorbent assay

To measure the secretion of CXCL1/KC and MCP-1 in mouse lung tissue, ELISAs were performed according to the manufacturer’s protocols using the appropriate ELISA kits (Mouse CXCL1/KC DuoSet ELISA, DY453-05, R&D Systems, and Mouse MCP-1 ELISA MAX™ Deluxe, 432704, BioLegend). Then, the optical density was spectrophotometrically measured at 450 nm using a microplate reader (Biotek Instruments, Inc., USA).

### Protein extraction and western blotting

Total proteins were extracted from lung tissue by lysis with RIPA buffer containing protease and phosphatase inhibitor cocktails (Beyotime, Shanghai, China). For neutrophils, the culture supernatant was solubilized in 5× concentrated sodium dodecyl sulfate-polyacrylamide gel electrophoresis (SDS-PAGE) sample buffer and boiled at 100 °C for 5 min. Protein concentrations were determined by the BCA method. Equal amounts of proteins were used for SDS-PAGE. After gel electrophoresis, the proteins were transferred into polyvinylidene difluoride membranes (Millipore, Billerica, USA). The membranes were blocked for 1 h in 5% skim milk at room temperature and incubated at 4 °C overnight with the following primary antibodies: anti-histone 3 (citrulline R2 + R8 + R17, ab5103, Abcam), anti-ZO-1 (ab216880), anti-occludin (ab216327), anti-E-cadherin (ab76055), and anti-GAPDH (5174s, Cell Signaling Technology). The membranes were then washed three times with TBST and followed by an incubation with horseradish peroxidase (HRP)-conjugated goat anti-rabbit IgG (EarthOx Life Sciences, CA, USA) or goat anti-mouse IgG (H + L) HRP (s0002, Affinity Biosciences) for 1 h at room temperature. GAPDH was used as the internal control. The antibody–antigen complexes were detected with Immobilon Western Chemiluminescent HRP Substrate (Millipore, MA, USA) and visualized using the G:Box gel doc system (Syngene, UK).

### Neutrophil isolation

Mouse bone marrow-derived neutrophils were isolated using a neutrophil isolation kit by following a protocol provided by the manufacturer (130-097-658, Miltenyi Biotec). The isolation procedure was conducted at 4 °C unless otherwise specified. In brief, mice were anesthetized and the animal surface was sprayed with 70% ethanol. The muscles were removed from both legs and the femur was separated from the tibia at the knee joint. The bone marrow cells were flushed using a sterile syringe filled with PBS containing 0.5% endotoxin-free BSA and 2 mM EDTA (magnetic-activated cell-sorting, MACS) and collected into a 50 ml conical tube through a 70 µm cell strainer. The bone marrow cells were collected by centrifugation at 500 × *g* for 5 min at 4 °C. The cell pellet was resuspended in 1 ml of RBC lysis buffer for ~5 min. The cell suspension was washed and centrifuged to collect the cells. The cell pellet was resuspended in 200 µl MACS buffer and 50 µl neutrophil biotin-antibody cocktail was added. Then, the cell suspension was mixed thoroughly and incubated for 10 min in the refrigerator at 4 °C. The cell suspension was washed and centrifuged to collect the cells. The cell pellet was resuspended in 400 µl MACS buffer and 100 µl antibiotin microbeads was added. The cell suspension was mixed well and incubated for 15 min in the refrigerator at 4 °C. The cell suspension was washed and centrifuged to collect the cells. The cell pellet was resuspended in 500 µl of MACS buffer. The cells were subsequently loaded onto a MACS buffer equilibrated LS column (Miltenyi Biotec) and washed LS column three times with 3 ml of MACS buffer. The cells through the LS column were harvested and allowed to warm up to room temperature in RPMI medium until they were used.

### NE-DNA quantification

The level of NE-DNA in the culture supernatant of neutrophils was measured by ELISA as described previously but with minor modifications^56^. In brief, a 96-well ELISA microplate was coated with ELA2 antibody (Proteintech, 17943-1-AP, 1:2000) and incubated at 4 °C overnight. After washing three times, the uncoated sites were blocked with 1% bovine serum albumin (BSA) in phosphate-buffered saline (PBS) at 37 °C for 1 hour. The microwells were again washed, and the samples were added to individual wells. The plate was incubated at 4 °C overnight. After washing, HRP-conjugated anti-DNA antibody (D5425-3-200, 1:100) was added to each well, and the plate was incubated at room temperature for 2 h. The plate was again washed, and TMB substrate was added. Absorbance was measured at 450 nm (end point) with a microplate reader (Biotek synergy) after the addition of the 2 N H2SO4 stop solution. Background absorbance values of the medium were subtracted.

### Flow cytometric assay for direct quantification of neutrophil extracellular traps

A flow cytometric assay was performed for NETs as described previously^57^. Briefly, neutrophils (1 × 10^6^) were seeded on a 24-well cell culture plate and incubated for 1 h in a CO_2_ incubator at 37 °C. Then, the cells were either left unstimulated or stimulated with 100 nM PMA or 1 μg/ml VP1 for 4 h. After the incubation, neutrophils were collected, fixed in 2% paraformaldehyde, blocked for 30 min with 5% goat sera without a permeabilization step, and incubated sequentially with the primary anti-histone H3 antibody (1:500, citrulline 2,8,17, ab5103, Abcam) at 4 °C for 1 h and with goat anti-rabbit IgG (H + L) Cross-Adsorbed Secondary Antibody, Alexa Fluor 555 (1:500, A-21428, Thermo) at 37 °C for 1 h in the dark. Each incubation was followed by a wash with 2% BSA in PBS and centrifugation at 4 °C for 5 min (2000 rpm). Samples were then analyzed by flow cytometry.

In a parallel experiment, prior to stimulation with PMA and VP1, neutrophils were pretreated with specific inhibitors for 30 min, including the protein arginine deiminase 4 (PAD4) inhibitor Cl-amidine (200 μM, Selleck) and the NADPH oxidase inhibitor DPI chloride (10 μM, Thermo).

### Confocal microscopy

Neutrophils (1 × 10^6^) were seeded on a sterile round glass cover slip that was placed in a 24-well cell culture plate. As described above in the flow cytometric assay for NETs, 100 μM PMA or 1 μg/ml VP1 was added. After 4 h of incubation, the glass cover slips with the attached cells were carefully removed from a 24-well culture plate and fixed with ice-cold acetone. Then, samples were blocked with 5% goat sera and stained overnight with a rabbit polyclonal antibody to histone 3 (citrulline R2 + R8 + R17) (1:300, ab5103, Abcam) and with a mouse anti-MPO antibody (1:300, ab90810, Abcam). The samples were washed in PBST and further stained with an Alexa Fluor® 555 goat anti-rabbit antibody (1:500, Life Technologies, USA) and an Alexa Fluor® 647 goat anti-mouse antibody (1:1000, Life Technologies, USA). Nuclei in the samples were stained with 4′6-diamidino-2-phenylindole. Images were captured by Zeiss LSM7 confocal fluorescence microscopes using the appropriate lenses and filters.

For experiments in vivo, the left lower lung of mice that were or were not treated with VP1 was carefully harvested, frozen in Tissue-Tek OCT media and prepared into 7-μm-thick slices. The fixation, blocking, antibody incubation, and image acquisition of these sections were performed as described at the cell level.

### Transepithelial electrical resistance (TEER) measurement

The TEER of the MLE-12 cell monolayer was measured using a Millicell® ERS-2 Electrical Resistance System. To eliminate the influence of temperature, the measurements were performed within 5 min after taking the culture plates out of the incubator. During this time, the samples did not show any reading drift. Before measurement, the electrodes were equilibrated and sterilized according to the manufacturer’s recommendations. Two hundred microliters of culture medium were added to the upper compartment of the cell culture system. The ohmic resistance of a blank (culture insert without cells) was measured in parallel. To obtain the sample resistance, the blank value was subtracted from the total resistance of the sample. The final unit area resistance (Ω cm^2^) was calculated by multiplying the sample resistance by the effective area of the membrane (0.3 cm^2^ for 24-well Millicell inserts).

### Live-cell imaging technique

Neutrophils (5 × 10^4^/well) were seeded onto a 96-well cell culture plate and treated with culture medium or VP1. Next, 1 μM SYTOX Orange (S11368, Thermo) was added before transferring the 96-well cell culture plate into the temperature- and CO_2_-controlled (37 °C, 5% CO_2_) environment of a Zeiss Cell Discoverer 7 microscope system. Live-cell phase-gradient contrast images of the individual field regions inside each well were automatically acquired using the ZEN Blue 2.3 software. Representative figure images were selected and additional image postprocessing steps (contrast adjustment, field selection, and scale bar addition) were performed in the Zeiss ZEN software.

### Statistics

Statistical analyses were performed using GraphPad Prism 7.0 software. Details of the individual tests are included in the figure legends. In general, data were tested for a normal distribution by the Kolmogorov–Smirnov normality test and were analyzed accordingly by an unpaired two-tailed *t*-test or the Mann–Whitney *U*-test. Data are presented as the mean ± SEM. In all cases, *P* values < 0.05 were considered significant.

## Supplementary information

S Movie 1 Blank Control

S Movie 2 VP1 promoted NETs
